# Protective Contributions against Invasive *Streptococcus pneumoniae* Pneumonia of Antibody and Th17-Cell Responses to Nasopharyngeal Colonisation

**DOI:** 10.1371/journal.pone.0025558

**Published:** 2011-10-07

**Authors:** Jonathan M. Cohen, Suneeta Khandavilli, Emilie Camberlein, Catherine Hyams, Helen E. Baxendale, Jeremy S. Brown

**Affiliations:** 1 Centre for Respiratory Research, Department of Medicine, University College London, London, United Kingdom; 2 Infectious Diseases and Microbiology Unit, Child Health Institute, University College London, London, United Kingdom; 3 Department of Immunology, Royal Free Hospital Medical School Campus, University College London, London, United Kingdom; Albany Medical College, United States of America

## Abstract

The nasopharyngeal commensal bacteria *Streptococcus pneumoniae* is also a frequent cause of serious infections. Nasopharyngeal colonisation with *S. pneumoniae* inhibits subsequent re-colonisation by inducing Th17-cell adaptive responses, whereas vaccination prevents invasive infections by inducing antibodies to *S. pneumoniae* capsular polysaccharides. In contrast, protection against invasive infection after nasopharyngeal colonisation with mutant *S. pneumoniae* strains was associated with antibody responses to protein antigens. The role of colonisation-induced Th17-cell responses during subsequent invasive infections is unknown. Using mouse models, we show that previous colonisation with *S. pneumoniae* protects against subsequent lethal pneumonia mainly by preventing bacteraemia with a more modest effect on local control of infection within the lung. Previous colonisation resulted in CD4-dependent increased levels of Th17-cell cytokines during subsequent infectious challenge. However, mice depleted of CD4 cells prior to challenge remained protected against bacteraemia, whereas no protection was seen in antibody deficient mice and similar protection could be achieved through passive transfer of serum. Serum from colonised mice but not antibody deficient mice promoted phagocytosis of *S. pneumoniae*, and previously colonised mice were able to rapidly clear *S. pneumoniae* from the blood after intravenous inoculation. Thus, despite priming for a Th17-cell response during subsequent infection, the protective effects of prior colonisation in this model was not dependent on CD4 cells but on rapid clearance of bacteria from the blood by antibody-mediated phagocytosis. These data suggest that whilst nasopharyngeal colonisation induces a range of immune responses, the effective protective responses depend upon the site of subsequent infection.

## Introduction

*Streptococcus pneumoniae* is the second commonest cause of fatal bacterial infection worldwide. Most deaths are due to pneumonia, which when severe is often associated with septicaemia. Nasopharyngeal colonisation with *S. pneumoniae* is nearly universal in infants, with carriage rates reaching 90% [1] but then rapidly falling to 10% in late childhood and adults [2]. *S. pneumoniae* pneumonia results from aspiration of colonising bacteria from the nasopharynx into the lungs. Infants and the elderly are particularly susceptible to *S. pneumoniae* pneumonia, causing an estimated 826,000 deaths annually in children under five years of age worldwide [3] and with an incidence of at least 50 per 100,000 in the elderly in developed countries [4]. *S. pneumoniae* septicaemia is also commoner in these age groups and has a high mortality [5]. Infants often have a primary septicaemia with no associated pneumonia, whereas in adults septicaemia usually develops as a complication of pneumonia. The reasons for the changing susceptibility to *S. pneumoniae* disease with age are not fully understood. Infants and the elderly are routinely vaccinated with capsular polysaccharide based vaccines, but these only protect against limited numbers of capsular serotypes and the unconjugated vaccine used in adults is not effective against pneumonia [6]. The conjugated vaccine used in children is effective but expensive, and has more limited serotype coverage so its efficacy could be reduced by vaccine induced changes in *S. pneumoniae* ecology. These limitations have stimulated interest in alternative *S. pneumoniae* vaccine strategies.

Although *S. pneumoniae* infections are common, the majority of colonised individuals do not develop disease suggesting there are robust natural mechanisms of immunity. These will include physical defences and innate immune responses [7], but the proportionally greater fall in *S. pneumoniae* disease rates compared to carriage rates after the first year of life suggests adaptive immune responses also have a role [1], [8]. *S. pneumoniae* colonisation in humans can induce anti-capsular antibodies, and by extension from vaccine data these were previously thought to be the main mechanism of naturally acquired adaptive immunity to invasive infection [8], [9]. However, in human models *S. pneumoniae* nasopharyngeal colonisation induces mainly anti-protein rather than anti-capsular antibody responses [10]. Furthermore, recent publications have shown that mice colonised with mutant strains of *S. pneumoniae*, including unencapsulated strains, develop anti-protein antibody responses [11]–[13]. Colonisation-induced protection was not seen in MHCII^−/−^ mice [12], suggesting an important role for CD4+ helper T-cells. CD4 T-cells may assist protective adaptive immune responses by providing T-cell help towards B-cell antibody production and/or through memory T-cell responses recalled during infectious challenge. Recall responses to bacterial antigens by human T-cells implies priming during natural exposure [14]. Such cells include Th17-cells, capable of producing large amounts of the cytokines IL-17A and IL-22 which facilitate cellular recruitment to sites of infection and enhance the release of antimicrobial products from local epithelium [15]. Th17 responses induced through immunisation can protect against challenge with other respiratory pathogens including *Bordetella pertussis* [16] and *Mycobacterium tuberculosis* [17]. *S. pneumoniae* nasopharyngeal colonisation of mice is known to elicit a Th17-cell response that assists primary clearance of *S. pneumoniae* from the nasopharynx and inhibits recolonisation [18], [19]. Furthermore, nasal immunisation with killed *S. pneumoniae* or purified pneumococcal proteins can also elicit a Th17-cell response capable of protecting against subsequent colonisation. Whether colonisation-induced Th17-cell responses are important for protective immunity against invasive disease such as *S. pneumoniae* pneumonia is not known.

We have used a murine model of nasopharyngeal colonisation with wild-type *S. pneumoniae* followed by pneumonia challenge to characterise the effects of colonisation on inflammatory and adaptive immune responses during subsequent infection, and to determine the relative contributions of antibody and Th17-cell mediated responses to protection.

## Methods

### Ethics statement

Experiments were approved by the UCL Biological Services Ethical Committee and the UK Home Office (Project Licence PPL70/6510). Experiments were performed according to UK national guidelines for animal use and care, under UK Home Office licence.

### Bacterial strains and culture conditions

*S. pneumoniae* D39 was a kind gift from James Paton, University of Adelaide [20]. Bacteria were cultured on Columbia agar with 5% horse blood or in Todd-Hewitt broth with 0.5% yeast extract in 5% CO_2_. Inocula for challenge experiments were prepared from mid-log phase cultures and stored at −70°C as single use aliquots.

### Colonisation and infection models

CBA/Ca inbred mice were obtained from Charles River UK Ltd. μMT mice were a kind gift from Dr Claudia Mauri, UCL, London (UK). Mice were colonised by instillation of 10^7^ cfu *S. pneumonia* D39 in 10 µl PBS into the nares under light halothane anaesthesia [12], [21], [22]. Control mice received 10 µl PBS alone. To obtain nasal washes the exposed trachea was flushed caudally with 200 µl PBS and the fluid exiting the nares collected. For the pneumonia challenge, 10^7^ cfu *S. pneumonia* D39 in 50 µl PBS was instilled into the nares under deep general halothane anaesthesia [21], [23], [24]. Animals were culled by exsanguination from the femoral artery under pentobarbital anaesthesia. BALF was collected by cannulating the exposed trachea and washing the airways three times serially with 1 ml sterile PBS. Lungs were collected aseptically after BAL had been performed into ice-cold PBS, minced and homogenised with sterile PBS as previously [21], [24]. For survival experiments, animals were monitored and culled when exhibiting previously defined features of terminal disease [23]. For intravenous challenge, mice received 5×10^6^ cfu *S. pneumoniae* D39 in 100 µl PBS via tail vein injection. Bacterial cfu were calculated by counting colonies after plating serial dilutions of target organ preparations on blood agar plates. Sera for cytokine and flow cytometry assays were kept at 4°C before being frozen and stored as single use aliquots at −70°C. For passive transfer experiments, naïve mice received 225 µl fresh pooled serum from colonised or control mice (obtained 28 days after colonisation) by intraperitoneal injection 6 h and 30 minutes prior to pneumonia challenge. For CD4+ cell depletion, mice received 250 µg mAb GK1.5 (eBiosciences) intraperitoneal 48 and 24 h preceding pneumonia challenge; flow cytometric examination of splenocytes confirming successful depletion of >90% of CD4 cells [19].

### Assessment of the inflammatory response to infection

To obtain BALF cell proportions, 300 cells from random fields of BALF cytospin preparations (Cytospin 3, Shandon) with rapid Romanowsky stain were counted according to standard morphological criteria, and absolute counts derived from haemocytometer cell counts. In selected experiments the degree of lung inflammation was assessed histologically. The entire left lung was fixed, processed to paraffin wax and stained with haematoxylin and eosin. Inflammation was assessed by a blinded observer using an established scoring system [24]. This involved assessing the percentage of overall lung involvement under ×10 magnification, followed by examination of 6 random fields under ×20 magnification. The composite score reported is the product of mean score for individual areas multiplied by percentage of lung affected. Sera and BALF supernatants cytokine levels were measured by ELISA (IL-17, IL-22 and IFN-γ, R&D Systems) or by Luminex™ bead immunoassay (Invitrogen) according to manufacturers' instructions.

### ELISAs for antibody responses

Antibodies specific to *S. pneumoniae* D39 antigens were measured by whole cell ELISA using established methods as previously described [25]. Briefly, *S. pneumoniae* D39 was grown to late log-phase, washed and resuspended in PBS to OD_580_ 1.0. 96-well plates were coated with this bacterial suspension, refrigerated overnight, then blocked with PBS 1% BSA prior to use. Sera were diluted in PBS 1% BSA before addition and binding to bacterial antigens detected with anti-mouse secondaries conjugated to alkaline phosphatase (Sigma).

### Flow cytometry assays of phagocytosis

Phagocytosis of FAMSE (Molecular Probes) labelled *S. pneumoniae* by freshly isolated human neutrophils (MOI of 10∶1) was measured using flow cytometry to obtain the MFI for cells associated with fluorescent bacteria as previously described [25]. *In vivo* phagocytosis by alveolar macrophages was measured using a flow cytometry method as previously described [26]. Briefly, mice were inoculated intranasally with FAM-SE labelled 10^7^ cfu D39, BALF obtained at 4 h and analysed using flow cytometry. Alveolar macrophages were identified based on scatter properties and bacterial phagocytosis measured as the MFI for macrophages associated with fluorescent bacteria.

### Statistics

Survival of challenged mice was compared by the log rank test. Bacterial loads in BALF and lung, antibody and cytokine levels (non-parametric) were compared by Mann-Whitney U-test. Presence or absence of bacteraemia was compared by the Fischer exact test. *In vivo* phagocytosis (parametric) were assessed by unpaired Student t-test. *In vitro* neutrophil phagocytosis was compared by one-way Anova with post-hoc tests. *P* values<0.05 were considered significant.

## Results

### *S. pneumoniae* colonisation model

**Figure 1:**
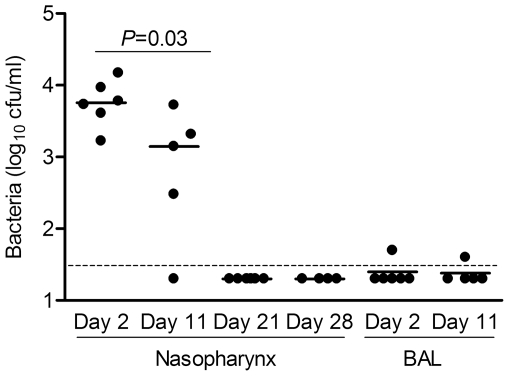
*S. pneumoniae* D39 colonises the nasopharynx of CBA/Ca mice. Bacterial cfu recovered from nasal washes and BALF 2, 11 and 28 days following intranasal inoculation in 10 µl of PBS with 10^7^ cfu D39 *S. pneumoniae*. Each dot represents results for an individual mouse and bars the median for each group. The dotted line is the limit of detection.

To establish nasopharyngeal colonisation, CBA/Ca mice were intranasally inoculated with 10^7^ cfu *S. pneumoniae* D39 suspended in 10 µl PBS and nasopharyngeal washes and bronchoalveolar lavage fluid (BALF) collected at 2, 11, 21 and 28 days to quantify *S. pneumoniae* colonisation. *S. pneumoniae* were recovered in nasopharyngeal washes from all mice at 2 days (median 5.68×10^3^ cfu/ml, interquartile range (IQR) 2.87–12.0), 80% of mice at 11 days (median 1.4×10^3^ cfu/ml IQR 0.16–3.66), but from no mice on days 21 and 28 (Fig. 1). Furthermore, no bacteria were recovered from homogenates of nasopharyngeal tissue harvested on day 21 (n = 6). On occasion a few *S. pneumoniae* colonies were recovered from BALF (≤50 cfu/ml), but no bacteria were recovered from lung homogenates or blood at any timepoint.

### Prior colonisation protects against lethal *S. pneumoniae* pneumonia by preventing bacteraemia

**Figure 2:**
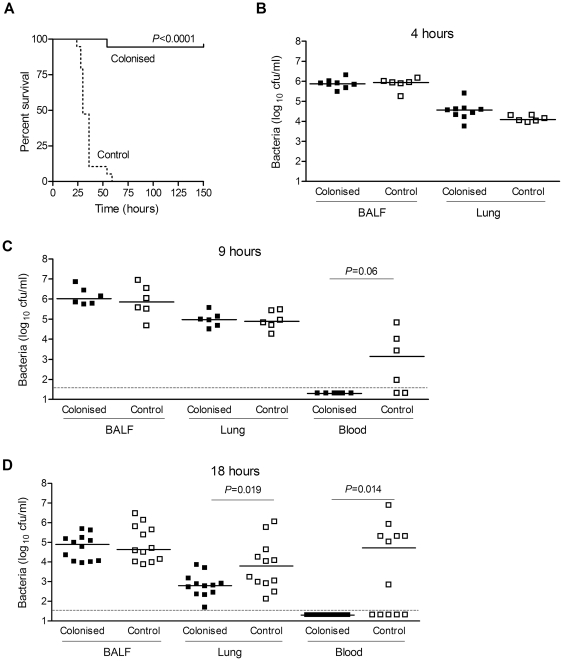
Effects of prior colonisation on progression of subsequent *S. pneumoniae* pneumonia. (A) Kaplan-Meier survival curves of previously colonised or control CBA/Ca mice following pneumonia challenge with 10^7^ cfu D39 *S. pneumoniae* in 50 µl PBS on day 28 (n = 18 or 19). (B–D) Bacterial cfu in BALF, lung or blood of colonised or control mice at (B) 4 h, (C) 9 h or (D) 18 h following pneumonia challenge. Each symbol represents results for an individual mouse and bars the median for each group. The dotted line is the limit of detection. Significance of the difference in bacterial numbers recovered from lungs of colonised and control mice was assessed by Mann-Whitney U-test. Significance of differences in presence or absence of bacteraemia was assessed by Fisher exact test. Data at 18 h are pooled from two experiments with similar results.

To investigate whether previous colonisation can induce adaptive mechanism(s) that protect against lethal infection, a model of rapidly developing fatal pneumonia was used in which mice were inoculated intranasally with 10^7^ cfu *S. pneumoniae* D39 suspended in 50 µl PBS. When challenged 28 days following colonisation, previously colonised mice were highly protected. All mice in the control group (which were sham colonised by intranasal administration of PBS) developed fatal infection with a median survival of 30 h whereas 94% of colonised mice survived (*P*<0.0001) (Fig. 2A). To further characterise colonisation-induced protection, groups of colonised or control mice given a day 28 pneumonia challenge were sacrificed at 4, 9 and 18 h to quantify the bacterial load in blood, BALF and lung (Fig. 2B–2D). In control mice there were significant numbers of bacteria in both BALF and lungs at all timepoints, and in the blood at 9 and 18 h. In colonised mice there were similar numbers of bacteria in BALF to controls at all timepoints and in the lungs at 4 and 9 h. By 18 h, there were approximately 1-log fewer bacteria in the lungs of previously colonised mice than controls. Strikingly, at both 9 and 18 h post-challenge no bacteria were recovered from the blood of colonised mice, demonstrating that protection against fatal pneumonia in colonised mice was associated with the prevention of detectable bacteraemia.

### Previous colonisation primes for greater mucosal and systemic IL-17 responses

**Figure 3:**
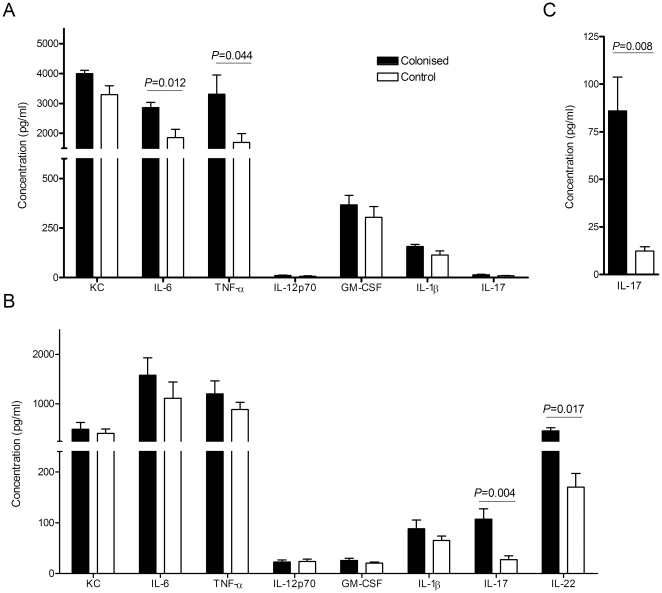
Effect of prior colonisation on cytokine levels during subsequent pneumonia. BALF samples were collected at either (A) 4 h or (B) 18 h and serum (C) at 18 h following pneumonia challenge of previously colonised (black bars) or control (white bars) CBA/Ca mice (n = 6 per group). Cytokine levels were measured by either Luminex bead assay or ELISA. Group mean + SEM are displayed. Dotted line represents limit of detection.

**Figure 4:**
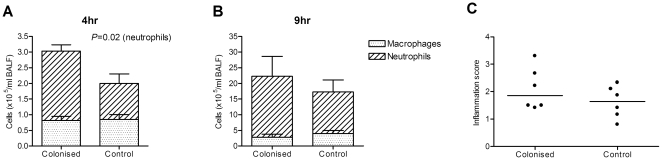
Effect of prior colonisation on inflammation during subsequent pneumonia. (A–B) Numbers of neutrophils (hatched region) and macrophages (dotted region) present in BALF of previously colonised or control CBA/Ca mice at (A) 4 h and (B) 9 h following pneumonia challenge. Bars represent mean + SEM (n = 6 mice per group). (C) Lung inflammation scores assessed by blinded histological examination of lung sections collected 18 h following challenge of colonised or control mice. Dots represent individual mice, bars represent medians.

To characterise the effect of previous colonisation on the inflammatory response during *S. pneumoniae* pneumonia, BALF cytokine levels were measured at 4 and 18 h after pneumonia challenge (Figure 3A, 3B). Compared to controls, in previously colonised mice there were modest but statistically significant increases in BALF levels of several innate cytokines, including TNF-α and IL-6, at 4 h but not at 18 h. In addition, at 18 h, IL-17 and IL-22 were markedly higher in BALF from previously colonised mice compared to controls, and IL-17 was also significantly higher in the serum (Figure 3C). Colonisation also primed for greater BALF neutrophil recruitment compared to controls at 4 h (mean 2.2±0.4×10^5^/ml versus 1.1±0.73×10^6^/ml, *P* = 0.02) but not at 9 h (mean 1.94±1.57×10^6^/ml versus 1.33±0.94×10^6^/ml, *P* = 0.43) (Fig. 4A, 4B). Hence, in previously colonised mice there was a modest increase in the strength of the early inflammatory response to subsequent *S. pneumoniae* lung infection and potentially significant mucosal and systemic Th17 responses. However, histological assessment of lung sections obtained 18 h after inoculation demonstrated there were no detectable differences in lung inflammation scores between colonised and control mice in established pneumonia (mean score 2.10±0.77 versus 1.61±0.59, *P* = 0.24) (Fig. 4C).

### CD4 cells were not required for colonisation induced protection against bacteraemia

**Figure 5:**
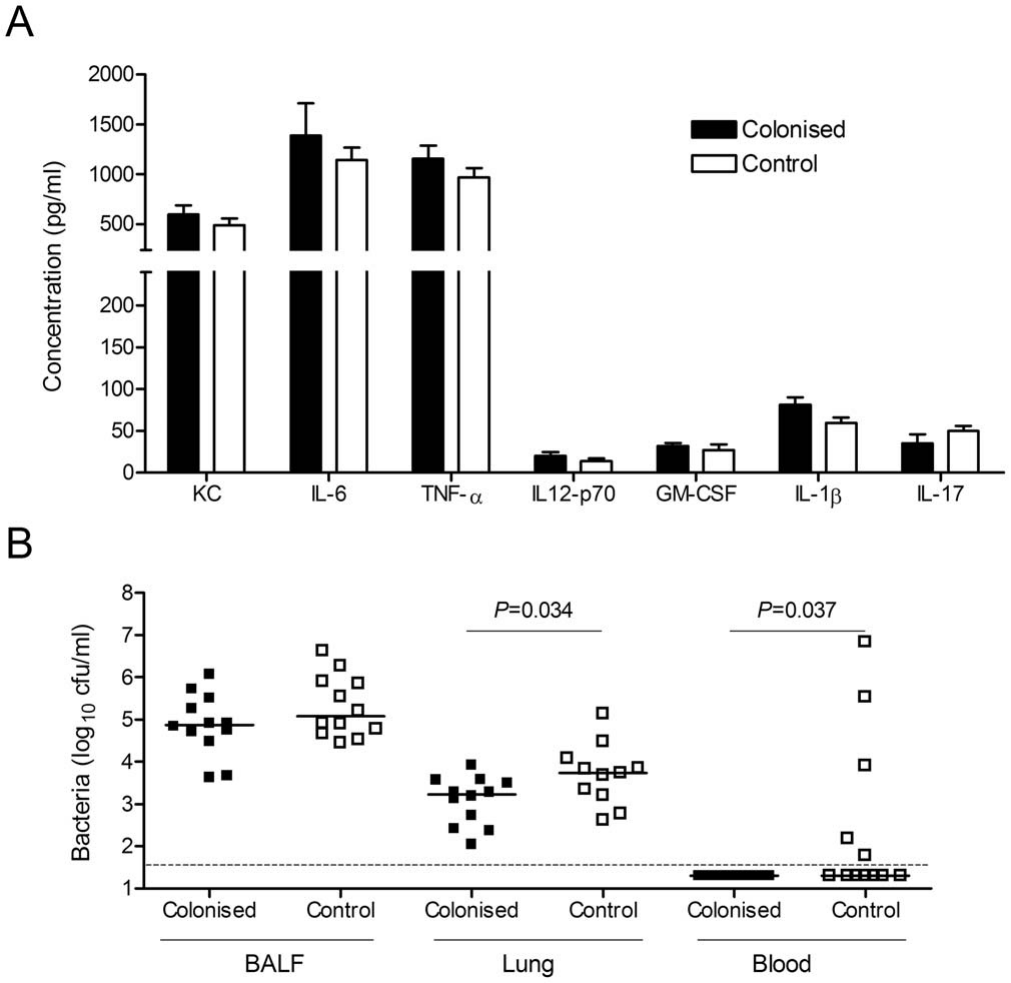
Role of CD4+ cells in the effect of prior colonisation on subsequent pneumonia. Cytokine levels (A) and bacterial CFU (B) recovered from colonised or control CBA/Ca mice 18 h following pneumonia challenge in the absence of CD4+ cells. Mice were depleted of CD4+ cells using GK1.5 prior to challenge. (A) Cytokine levels in BALF of colonised mice (black bars) or control mice (white bars) measured by Luminex or ELISA. Group mean + SEM are displayed. (B) Bacterial cfu in BALF, lung and blood of colonised or control CBA/Ca. Each symbol represents results for an individual mouse and bars the median for each group. The dotted line is the limit of detection. Significance of the difference in bacterial numbers recovered from lungs of colonised and control mice was assessed by Mann-Whitney U-test. Significance of differences in presence or absence of bacteraemia was assessed by Fisher exact test. Data are pooled from two experiments with similar results.

Th17-cell mediated immunity assists nasopharyngeal clearance of *S. pneumoniae* [19], and the enhanced IL-17 response to pneumonia in previously colonised mice suggest a Th17 response might be contributing towards protection against invasive disease. To investigate this possibility, colonisation and subsequent *S. pneumoniae* pneumonia challenge experiments were repeated in mice depleted of CD4+ cells immediately before challenge using anti-CD4 antibody. CD4 depletion (>90% efficacy) abolished the colonisation-induced enhancement of BALF IL-17 levels at 18 hours in response to *S. pneumoniae* pneumonia in previously colonised mice (Figure 5A). Furthermore, in the absence of CD4 cells, no IL-17 was detected in the serum following infection of either colonised or control mice (data not shown). These data suggest that colonisation-induced Th17-cells were the source of the enhanced mucosal and systemic IL-17 responses observed in colonised mice.

In the absence of CD4 cells, 42% of challenged control mice developed significant bacteraemia by 18 h following challenge (Figure 5B). Nevertheless, even in the absence of Th17 cell responses, colonised mice were still fully protected against bacteraemia (*P* = 0.037). In addition, there were still fewer bacteria in the lungs of colonised than control mice despite the absence of CD4 T-cells. Thus, the enhanced CD4-cell dependant IL-17 response to subsequent pneumonia in colonised mice does not seem to be required for protection in this model.

### Colonisation induces a protective antibody response

**Figure 6:**
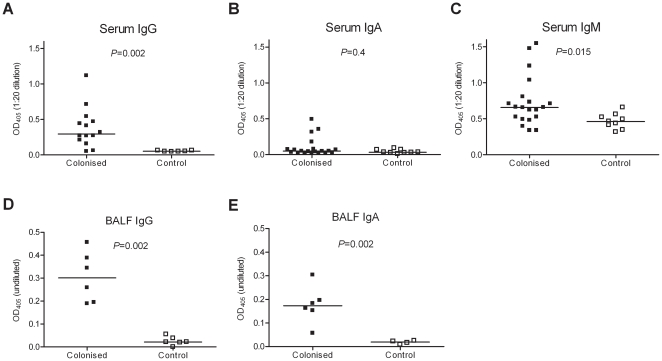
Systemic and mucosal antibody responses to colonisation. IgG (A and D), IgA (B and E) and IgM (C) responses to D39 in serum (A–C) or BALF (D–E) obtained from colonised or control mice measured using whole cell ELISAs.

The continued colonisation-induced protection in CD4 depleted mice demonstrated there must be alternative mechanisms of protection to Th17 responses, suggesting a role for colonisation-induced antibody. We therefore assessed antibody responses to *S. pneumoniae* colonisation in our model. Whole cell ELISAs detected high levels of serum IgG against D39 antigens in nearly all colonised mice 28 days after colonisation (Fig. 6A). Only a small number of colonised mice had serum IgA or IgM responses greater than controls (Fig. 6B, 6C). There were strong IgG and IgA responses to D39 evident in BALF (Fig. 6D, 6E).

**Figure 7:**
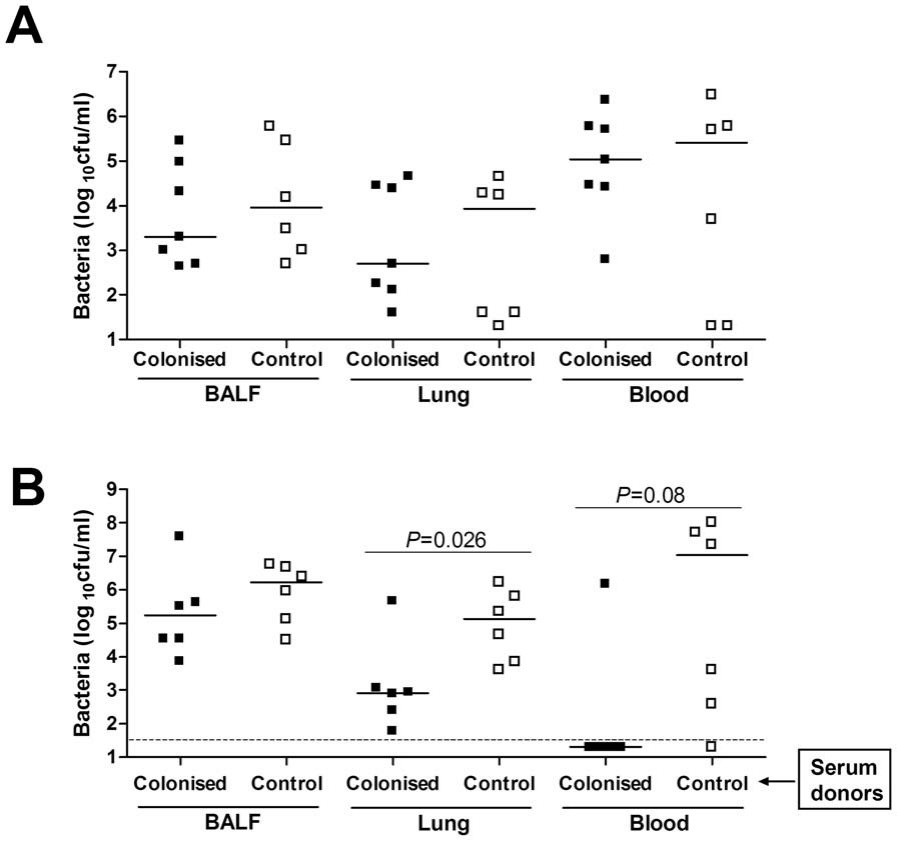
Role of antibody in the effect of prior colonisation on subsequent pneumonia. (A) Bacterial cfu in BALF, lung and blood of colonised or control μMT mice 18 h following pneumonia challenge. (B) Bacterial cfu in BALF, lung and blood of passively immunised CBA/Ca mice 18 h following pneumonia challenge. Mice received pooled serum collected from either colonised or control CBA/Ca mice prior to challenge. Each symbol represents results for an individual mouse and bars the median for each group. The dotted line is the limit of detection. Lung cfu are compared between groups by Mann-Whitney U-test. Presence or absence of bacteraemia was assessed by Fisher exact test.

To determine whether protection was dependant on the presence of antibody, further colonisation and challenge experiments were performed in μMT mice which congenitally lack antibody. All colonised μMT mice developed bacteraemia by 18 h following challenge (Fig. 7A). To identify whether the antibody response to colonisation was sufficient to protect against subsequent pneumonia, naïve CBA/Ca mice were passively vaccinated by intraperitoneal injection with fresh pooled sera obtained from colonised or control CBA/Ca mice, then given a pneumonia challenge. The anti-D39 whole cell IgG titre of donor serum was 1/2260, and achieved a mean titre in the serum of passively immunised mice of 1/309±1/110. Compared to recipients of control serum, passively immunised mice had significantly fewer bacterial CFU recovered from the lungs at 18 h. Furthermore, there was a strong trend towards prevention of bacteraemia, with only 1 of 6 recipients of immune serum developing bacteraemia compared to 5 of 6 recipients of control serum by 18 h post-pneumonia challenge (Fig. 7B), similar to the data obtained with actively colonised mice (Fig. 2B–2D). Overall, these data demonstrate that the systemic antibody response to *S. pneumoniae* colonisation is both necessary and sufficient to protect, and that whilst colonisation induces a Th17 cell response, this is not required.

### Enhanced *S. pneumoniae* phagocytosis in serum from previously colonised mice

**Figure 8:**
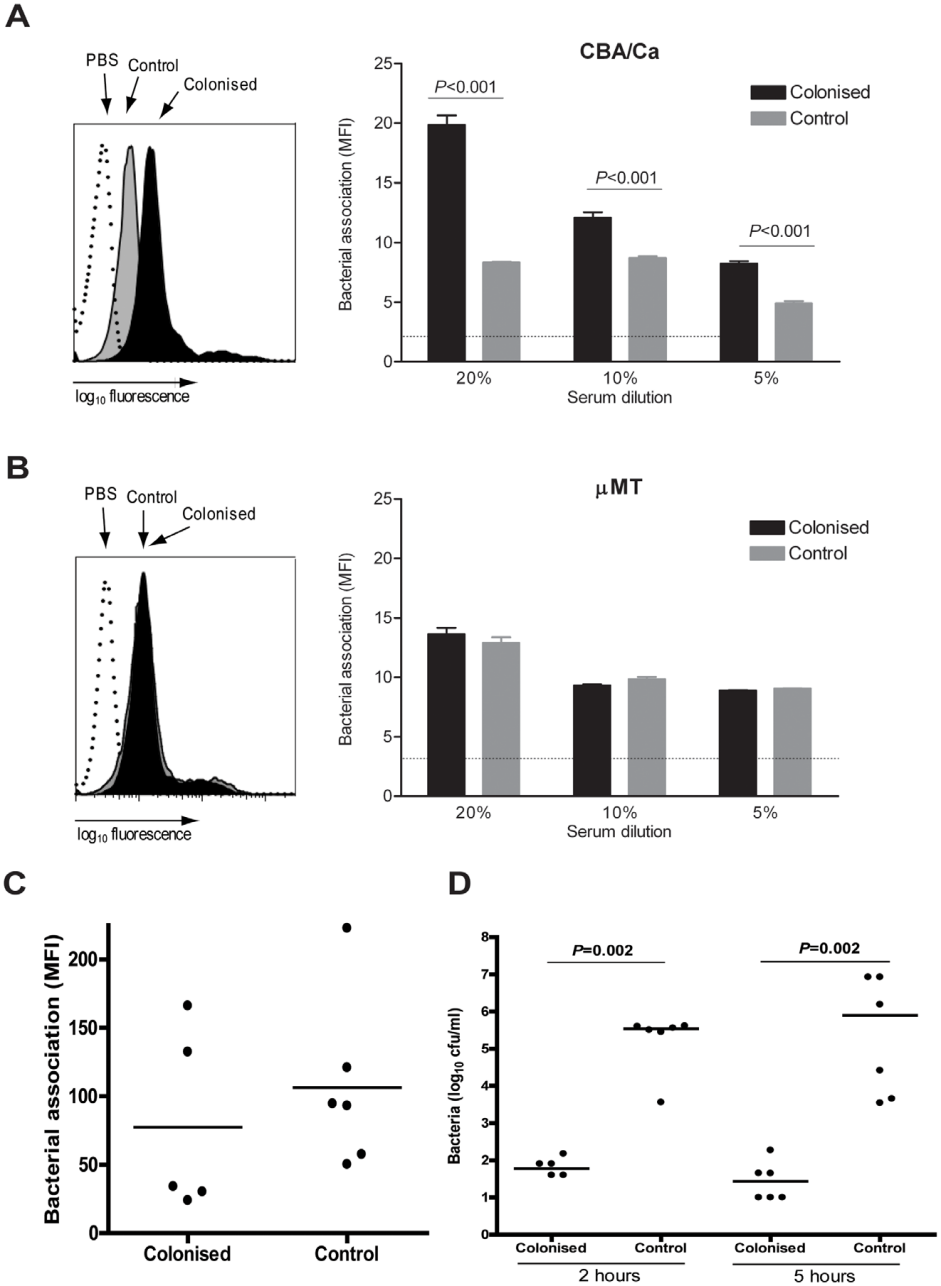
Colonisation induces serum opsonophagocytic antibody. (A–B) Flow cytometry assays of neutrophil phagocytosis of FAM-SE labelled D39 after incubation in serum from either (A) colonised or control CBA/Ca mice or PBS, or (B) colonised or control μMT mice or PBS. A representative histogram is shown on the left (clear PBS, black 20% control sera, grey 20% colonised sera), and mean MFI ± SEM (n = 4 replicates) for each condition in the graph on the right. (C) MFI of alveolar macrophages present in BALF of previously colonised or control CBA/Ca mice 4 h following challenge with 10^7^ cfu FAM-SE-labelled D39 *S. pneumoniae*. Dots represent individual mice, bars represent medians. (D) Numbers of cfu of *S. pneumoniae* recovered from blood of colonised or control mice inoculated intravenously with 5×10^6^ cfu *S. pneumoniae* D39 (n = 6 per group) at 2 and 5 h. Dots represent data from individual mice, bars represent group medians.

To further characterise the mechanism of antibody mediated protection in colonised mice, flow cytometry assays were used to assess the impact of colonisation-induced serum antibody on neutrophil association with live *S. pneumoniae*. Incubation of 6-carboxyfluorescein succinimidyl ester (FAMSE) labelled D39 with serum from colonised CBA/Ca mice resulted in enhanced association with human neutrophils (known to be mainly due to phagocytosis [27] compared to serum from control CBA/Ca mice (Fig. 8A). No differences in phagocytosis were observed when bacteria were incubated in sera from colonised or control μMT mice (Fig. 8B), confirming that serum antibody from colonised CBA/Ca mice bound to the *S. pneumoniae* surface was responsible for the enhanced phagocytosis in this serum. In contrast, when colonised and control mice were challenged with FAM-SE-labelled D39 there were no differences in the association of fluorescent *S. pneumoniae* with alveolar macrophages recovered from BALF 4 h post-inoculation (Fig. 8C). Hence colonisation resulted in improved antibody dependent phagocytosis in serum but had no detectable effect on alveolar macrophage phagocytosis *in vivo*. This was consistent with prior colonisation having no effect on the number of bacteria recovered from BALF following pneumonia, but marked protection against pneumonia-associated bacteraemia (Fig. 2B–2D).

To investigate whether previous colonisation improved systemic clearance of *S. pneumoniae*, known to be largely dependent on phagocytosis [28], mice were inoculated intravenously with 5×10^6^ cfu of D39. At 2 and 5 h post-intravenous inoculation there were over 10^4^ fewer bacteria in the blood of previously colonised mice compared to control mice (Fig. 8D), demonstrating that colonisation promoted rapid clearance of *S. pneumoniae* from the blood consistent with previous colonisation resulting in a marked augmentation of serum-mediated phagocytosis.

## Discussion

Recent data obtained using mouse models of colonisation have emphasised the importance of Th17 responses for protection against subsequent re-infection of the nasopharynx, and have shown that colonisation also protects against subsequent invasive infection. The development of protection against invasive infection required both CD4 cells and antibody but the precise mechanisms involved have not been clearly defined [12]. In this study, we have investigated in detail potential mechanisms of protection against subsequent pneumonia associated with *S. pneumoniae* nasopharyngeal colonisation. Mortality due to *S. pneumoniae* is mainly related to severe infections with septicaemia. Hence, to identify protective mechanisms that are effective against severe infection we have deliberately chosen a disease model of fulminant infection requiring a large inoculum with rapid spread of bacteria from the lungs to the blood and a high mortality. Several mechanisms through which colonisation may impact on subsequent disease progression were identified.

Firstly, prior nasopharyngeal colonisation was associated with a stronger mucosal inflammatory response 4 h after pneumonia challenge, with higher levels of some cytokines and a more rapid influx of neutrophils. The rapidity of onset of this difference between colonised and control mice suggests that colonisation may lead to alteration of the ‘innate immune rheostat’, priming for a more robust response to subsequent pneumonia challenge [29]. During the time that the nasopharynx remains colonised, small numbers of bacteria are likely to be aspirated into the lungs. These could have effects on innate immune cells such as macrophages and gamma-delta T-cells as shown for non-bacteraemic pneumonia [30]. This could lead to more robust cytokine production early in subsequent infection. Alternatively, mucosal antibody induced through prior colonisation may facilitate the interaction between bacteria and host cells such as alveolar macrophages during early pneumonia challenge and enhance cytokine responses. However, the effects of prior colonisation on the early inflammatory response in the lungs did not limit the development of disease in this model. There were no significant differences between colonised and control mice in *in vivo* phagocytosis of bacteria by alveolar macrophages, in bacterial CFU in BALF or lung, or in the histological severity of pneumonia. Potentially, the effects of colonisation on early inflammatory responses may have protective effects with other less fulminant models of pneumonia using lower inocula and / or less virulent *S. pneumoniae* strains.

Nasopharyngeal colonisation also primed for an enhanced lung and systemic IL-17 response following *in vivo* challenge. This was dependant on CD4-cells, strongly suggesting that it derives from a Th17-cell response to colonisation similar to that found in the nasopharynx following re-colonisation [19], [31]. However in contrast to the importance of a Th17-cell response for preventing re-colonisation of the nasopharynx, colonised mice depleted of CD4 cells were still protected against bacteraemia and still had reduced lung CFU at 18 hours. Hence, Th17 responses are not required for colonisation-induced protection in this model. The inability of previous colonisation to protect MHCII deficient mice against lethal challenge [12] is likely to reflect a need for CD4 T-cells in supporting the development of a mature antibody response to colonisation, rather than an effector role at the time of challenge. Rapid neutrophil recruitment occurs within the lungs even in uncolonised control mice, and bacteraemia is established relatively early in infection, probably eclipsing any benefits from a Th17-cell mediated increase in phagocyte recruitment. In contrast, recruitment of phagocytes to the nasopharynx during *S. pneumoniae* colonisation is delayed unless supported by an adaptive Th17 cell response induced by a previous colonisation event [19]. Hence a Th17 response is critical for adaptive immunity against colonising *S. pneumoniae* but not for the rapidly invasive model of pneumonia described in this mansucript.

In addition, there is some evidence that Th17 cells actually might be deleterious during *S. pneumoniae* pneumonia [32], [33], possibly explaining why we found CD4 depletion was associated with a trend towards fewer bacteria in the blood at 18 h. Vaccination-induced Th17-cell responses may still be beneficial for protection against a less fulminant pneumonia in which bacteria remain within the lung.

The strongest effect of colonisation observed during challenge in our model was the protection against bacteraemia. This suggests protective responses either prevent spread of *S. pneumoniae* from the lungs to the blood or that on reaching the blood bacteria are rapidly cleared. Nearly all mice developed serum IgG responses to *S. pneumoniae* D39 antigens, and experiments with antibody-deficient mice demonstrated that protection against bacteraemia was highly dependent on antibody to *S. pneumoniae*. Following passive transfer of serum by intraperitoneal injection, there was a significant reduction in the number of bacteria present in the lungs 18 h following infection. This suggests that systemic antibody can assist control of infection within the lungs as well. Antibody to *S. pneumoniae* is mainly thought to cause protection by opsonising bacteria for phagocytosis, and *in vitro* phagocytosis assays and *in vivo* IV clearance data confirmed that colonisation induced antibody responses improved *S. pneumoniae* phagocytosis. The strength of the effect on IV clearance was particularly striking, reducing bacterial CFU by a factor of 3 to 4 log_10_, readily explaining why colonised mice do not develop septicaemia. The observation that colonisation prevents the development of systemic infection provides one potential explanation for the rapid fall in the incidence of *S. pneumoniae* septicaemia in older children after a period of recurrent colonisation as infants.

Which *S. pneumoniae* antigens are the targets for protective responses in our model is not clear. Both capsular and protein antigens may induce antibody responses after murine nasopharyngeal colonisation [11], [13]. However, colonisation with *S. pneumoniae* mutant strains not able to express the capsule or seemingly immunodominant protein antigens were still able to induce protective responses [13] suggesting antigens inducing protective antibody responses are partially redundant. We are currently identifying which antibodies dominate the immune response to colonisation in this model, and which are the important antibodies in effecting protection.

To conclude, we have investigated the mechanisms of protection against subsequent pneumonia induced by nasopharyngeal colonisation with *S. pneumoniae*. In our model although colonisation results in a more rapid inflammatory response during early lung infection and a significant CD4-dependent IL-17 response, neither was necessary for the powerful protection against fulminant pneumonia provided by prior colonisation. Instead, protection was due to serum antibody responses that promoted rapid clearance of *S. pneumoniae* from the blood.
